# Resistance components of liquid thermoelectric converter composed of [Fe(CN)_6_]^3−^/[Fe(CN)_6_]^4−^ aqueous electrolyte and graphite-dispersing electrodes

**DOI:** 10.1039/d5ra07066j

**Published:** 2025-11-06

**Authors:** Soshi Fukuda, Yutaka Moritomo

**Affiliations:** a Graduate School of Pure & Applied Science, University of Tsukuba Tennodai 1-1-1, Ibaraki Tsukuba 305-8571 Japan moritomo.yutaka.gf@u.tsukuba.ac.jp; b Faculty of Pure & Applied Science, University of Tsukuba Tennodai 1-1-1, Ibaraki Tsukuba 305-8571 Japan; c Tsukuba Research Center for Energy Materials Science (TREMS), University of Tsukuba Ibaraki Tsukuba 305-8571 Japan

## Abstract

Liquid thermoelectric converter (LTE) converts environmental heat into electric power *via* the electrochemical Seebeck coefficient *α*. Here, we systematically investigated components of device resistance *R*, *i.e.*, solute (*R*_s_), charge-transfer (*R*_ct_), and diffusion (*R*_dif_) resistances, of LTE composed of 0.8 M Na_4_[Fe(CN)_6_]/K_3_[Fe(CN)_6_] aqueous electrolyte and graphite-dispersing electrodes. *R*^−1^_ct_ and *R*_dif_^−1^ linearly increase with *t* in the thin *t* region (*t* ≤ 40 μm) reflecting the increase in electrochemical active surface area (EASA). The maximum power *W*_max_ of the ferro/ferri LTE (*t* = 97 μm) reaches 0.76 W m^−2^ at Δ*T* = 50 K, suggesting the effectiveness of the graphite-dispersing electrode in ferro/ferri LTE.

## Abstract 2

The electrochemical impedance spectroscopy of ferri/ferro LTE revealed that the mass transfer process is the main rate-limiting factor (*R*_dif_ ≫ *R*_ct_).

## Graphical abstract

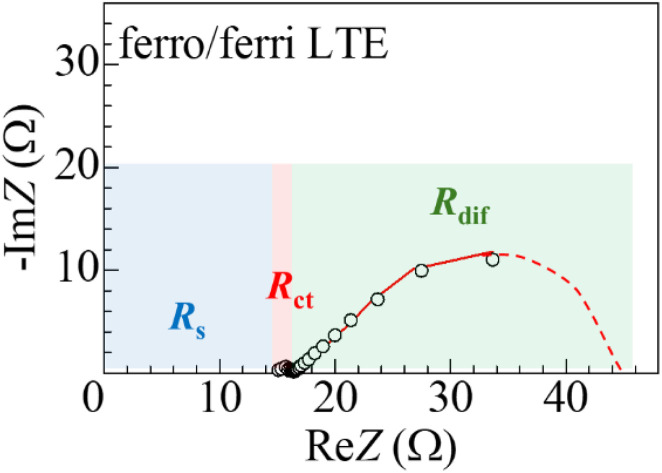

## 1 Introduction

Recently, energy harvesting devices are intensively investigated from the viewpoint of achieving sustainable development goals (SDGs). Among the devices, liquid thermoelectric converter (LTE)^1–5^ has simple device structure and is composed of electrolyte containing a redox couple and two identical electrodes. In addition, LTE is composed of low cost materials and is suitable for a widely used energy harvester. The LTE utilizes the thermogalvanic effect (electrochemical Seebeck coefficient *α*) at each electrode to convert temperature differences Δ*T* between the electrodes into the electromotive force *V*. The research of LTE is increasingly active with significant progresses.^6–25^ The performance of LTE is determined by *α*, electric conductivity *σ*, and thermal conductivity *κ*. The maximum (*W*_max_) of power is expressed as 
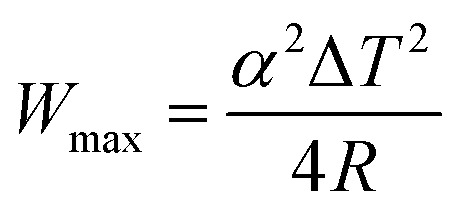
, where Δ*T* and *R* is the temperature difference between the electrodes and device resistance, respectively. To increase *W*_max_, it is necessary to reduce *R* or increase *α*. *R* can be decomposed into solution resistance *R*_s_, charge transfer resistance *R*_ct_, and diffusion resistance *R*_dif_.^26^

The LTE composed of aqueous solution containing [Fe(CN)_6_]^4−^/[Fe(CN)_6_]^3−^ (ferro/ferri LTE) or Fe^2+^/Fe^3+^ (Fe^2+^/Fe^3+^LTE) is extensively investigated because the solution is stable and exhibits rather high *α*. *α* is −1.42 mV K^−1^ for ferro/ferri LTE and 1.76 mV K^−1^ for Fe^2+^/Fe^3+^LTE.^9^ Kim *et al.*^11^ reported that addition of organic solvent in ferro/ferri LTE significantly increases *α*. Tanaka *et at.*^17^ performed a systematic investigation on the additive effect in ferro/ferri LTE and ascribed the effect to precipitation of K_4_[Fe(CN)_6_]. On the other hand, Yu *et al.*^8^ reported that addition of guanidinium in ferro/ferri LTE significantly increases *α* at 0.4 M from −1.4 mV K^−1^ to – 3.73 mV K^−1^. Buckingham *et al.*^21^ demonstrated that *α* of Fe^2+^/Fe^3+^ LTE can be optimized by pH of the electrolyte. Li *et al.*^25^ reported that addition of HClO_4_ significantly increases *α* and *σ* in Fe^2+^/Fe^3+^ LTE. They interpreted the enhancement in terms of the formation of hexa-aqua iron (III/II) complexes *via* variation of pH of the electrolyte.

To realize practical applications in society, it is necessary not only to increase *W*_max_ but also to reduce the costs of manufacturing elements. The coated electrodes used in ion secondary batteries are not only low cost but can also be made large and mass-produced by utilizing printing technology. Recently, Aiba *et al.*^27^ demonstrated that graphite-dispersed coated electrodes significantly enhance *W*_max_ of Fe^2+^/Fe^3+^ LTE. They further investigated resistance components against electrode thickness *t* and found that *R*_ct_^−1^ and *R*_dif_^−1^ linearly increase with *t* in the thin *t* region (*t* ≤ 80 μm). Here, we will investigate the effectiveness of the graphite-dispersing electrode for the ferro/ferri LTE.

*R* of LTE can be decomposed into *R*_s_, *R*_ct_, and *R*_dif_.^26–28^*R*_s_ is proportional to the electrode distance *d* because it is determined by the balance between the electrostatic force and frictional force acting on a moving ion.^29^*R*_ct_ is governed by the redox reaction rate *k* at the electrode surface and is independent of *d*. *k* is expressed as 
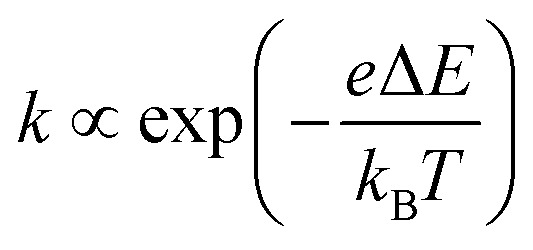
, where Δ*E* (= *E* – *E*_eq_; *E* and *E*_eq_ are the electrode and equilibrium potentials, respectively) and *k*_B_ are the overpotential and Boltzmann constant, respectively. *k* is proportional to the electrochemical active surface area (EASA), which is usually evaluated by the magnitude of the electric double layer capacitance *C*_d_.^31–33^ In the region of 
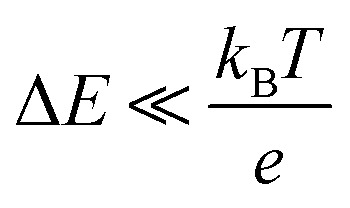
, the charge-transfer current *J*_ct_ is expressed as 
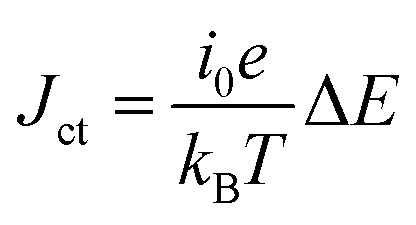
,^26^ where *i*_0_ is the exchange current. On the other hand, *R*_dif_ is governed by diffusion of reactants and/or products driven by the concentration gradient within the diffusion layer. When a constant current is applied to a LTE, the diffusion layer gradually forms due to the redox reaction at the electrode surface. In other words, *R* is essentially *R*_s_ + *R*_ct_ immediately after the current apply, because there exits neither diffusion layer nor diffusion. When enough time has passed, the system reached a steady state with a steady diffusion layer, which drives mass transfer equal to the redox reaction rate at the electrode surface. In the steady state, *R* is expressed as *R*_s_ + *R*_ct_ + *R*_dif_. In other words, *R*_dif_ can be evaluated by subtracting *R*_s_ + *R*_ct_ from *R* in the steady state. Importantly, *R*_dif_ is directly related to the finite diffusion impedance 
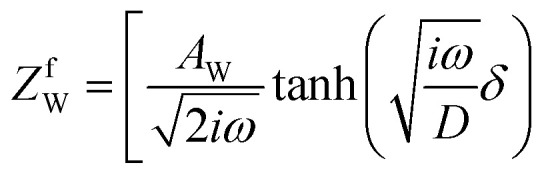
; where *A*_W_, *ω*, *δ*, and *D* are the Warburg coefficient, angular velocity, thickness of the diffusion layer, and diffusion constant, respectively].^30^ At low frequency limit (*ω* → 0), *Z*^f^_W_ converges a constant value 
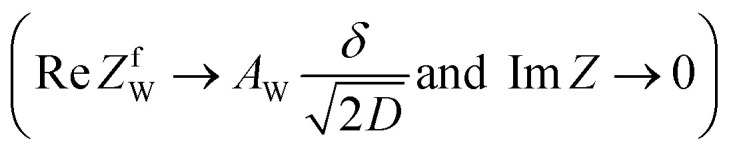
. *R*_dif_ corresponds to the low frequency limit of 
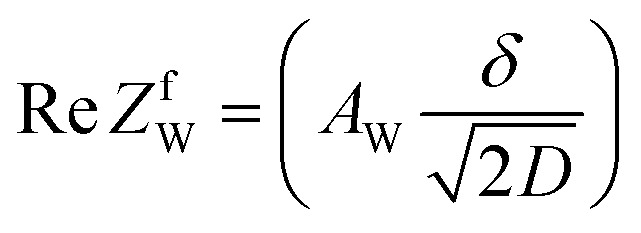
.

In this paper, we investigated the resistivity components in ferro/ferri LTE composed of graphite-dispersing electrodes against *t*. In particular, *R*_dif_ was evaluated using two methods, *i.e.*, subtraction of *R*_s_ + *R*_ct_ from *R* and analysis by Randles equivalent circuit including *Z*^f^_W_. Low frequency electrochemical impedance spectroscopy (EIS) reveals that mass transfer process is the main rate-limiting factor in ferro/ferri LTE.

## 2 Experimental section

### 2.1 Coated electrode and electrolyte

The graphite-dispersing coated electrode was prepared as described in literature.^27,28^ The graphite powder and polyvinylidene difluoride (PVDF) were mixed thoroughly in a weight ratio of 9 : 1 with *N*,*N*-dimethylformamide (DMF). Graphite powder (CAS RN: 7782-42-5, Wako Special Grade) was purchased from FUJIFILM Wako corp. and used as received. The mixture was coated onto a stainless-steel foil (SUS304, 10 μm) with use of an applicator, and was dried in vacuum at 60 °C. The electrode thickness *t*, which was controlled by the height of the applicator, was evaluated with a digital micrometer.

The electrolyte was aqueous solutions containing 0.8 M Na_4_[Fe(CN)_6_] and 0.8 M K_3_[Fe(CN)_6_]. The reason for choosing Na_4_[Fe(CN)_6_] rather than K_4_[Fe(CN)_6_] is the higher solubility of the former. The solutes were purchased from FUJIFILM Wako corp. and used as received. The solute concentration (=0.8 M) was the same as in the literature,^27^ because *W*_max_ of aqueous LTE increases with solute concentration.^9^

### 2.2 Fabrication of LTE

Resistance components and output characteristics of LTE were investigated with a specially designed two-pole cell.^34^ The cell consists of a cylindrical electrolyte tank and two aluminum bases. The electrolyte tank was a 7.3 mm *ϕ* polytetrafluoroethylene (PTFE) cylinder, whose ends were sealed with the bases. The inner surfaces of the bases were completely covered with the graphite-dispersing electrodes or commercially available graphite sheets (GS; *t* = 220 μm). The electrode distance *d* and area *s* were 10 mm and 42 mm^2^, respectively. Temperatures of the high (*T*_H_) and low (*T*_L_) electrodes were monitored with T-type thermocouples and independently controlled with a Peltier element equipped with a heat sink and a cooling fan, which were attached to the outer surfaces of the bases. Δ*T* is defined by Δ*T* = *T*_H_ – *T*_L_.

### 2.3 Device resistance and resistance components

Device resistance *R* was evaluated from current *I*–voltage *V* relation at 298 K (Δ*T* = 0 K). *I* was changed stepwisely. The *V* value at each *I* was measured after waiting several minutes until the time change of *V* almost disappeared. Measurements were performed with horizontally orientated LTE. The slope of the *I*–*V* plot corresponds to *R*.

**Fig. 1:**
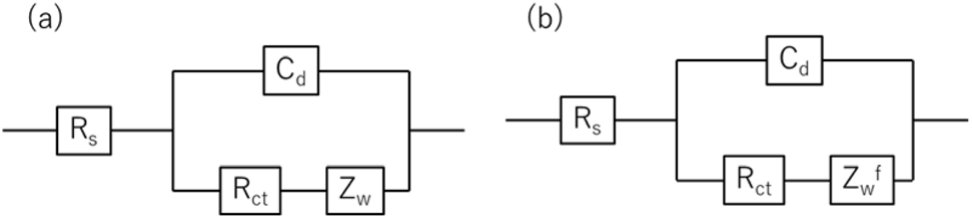
(a) Randles equivalent circuit composed of *R*_s_, *R*_ct_, *C*_d_, and *Z*_W_. (b) Randles equivalent circuit composed of *R*_s_, *R*_ct_, *C*_d_, and *Z*^f^_W_.

Resistance components, *i.e.*, *R*_s_, *R*_ct_ and *R*_dif_, were evaluated by electrochemical impedance spectroscopy (EIS) with use of a potentiostat (Vertex.one.EIS, Ivium technologies) at 298 K (Δ*T* = 0 K). The whole measurements were performed with horizontally orientated LTE. No diffusion layer is formed in the fast *f* region (*f* ≥ 1Hz) while its effects begin to appear in the slow *f* region (∼mHz). In the usual case, the frequency *f* of the alternative electric field was from 1 Hz to 20 kHz and the amplitude was 10 mV. The obtained EIS data (*f* ≥ 1Hz) were analyzed with a Randles equivalent circuit composed of *R*_s_, *R*_ct_, *C*_d_, and Warburg impedance 
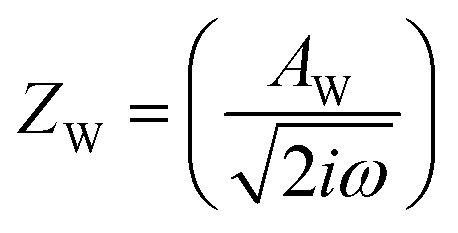
, as shown in Fig. 1(a). *R*_dif_ was evaluated by subtracting *R*_s_ + *R*_ct_ from *R*. Hereafter, thus evaluated value will be referred to as 
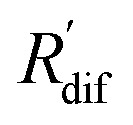
 to distinguished it from *R*_dif_ evaluated directly by EIS measurements.

To evaluate *R*_dif_ from EIS measurements, we further performed EIS measurement in an expanded *f* range from 1 m Hz to 25 kHz at 298 K. The amplitude was 10 mV. The obtained EIS data (≥1 mHz) were analyzed with a Randles equivalent circui composed of *R*_s_, *R*_ct_, *C*_d_, and *Z*^*f*^_W_, as shown in Fig. 1(b). At low frequency limit (*ω* → 0), *Z*^f^_W_ converges a constant value 
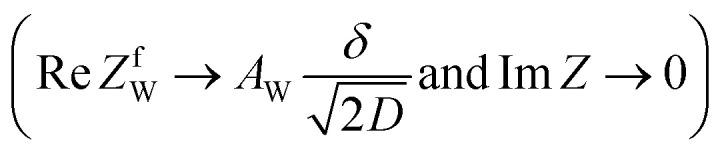
. *R*_dif_ corresponds to the low frequency limit of 
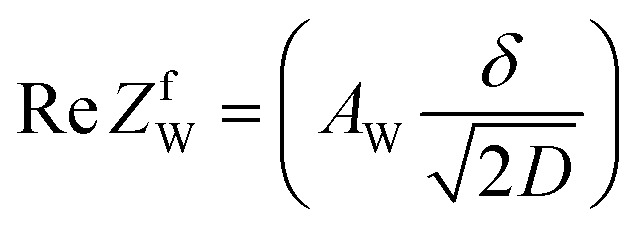
.

### 2.4 Output characteristics of LTE

Output characteristics of LTE were investigated at *T*_L_ = 300 K and *T*_H_ = 330 K (Δ*T* = 30 K). The *V* value was measured against current *I*. *I* was changed stepwisely. The *V* value at each *I* was measured after waiting a few minutes until the time change of *V* almost disappeared. The power *W* is expressed as *W* = IV. The open-circuit voltage *V*_0_ and *R* were evaluated by least-squares fitting of the *I*–*V* plot with *V* = *V*_0_ + *IR*. *W*_max_ is expressed as 
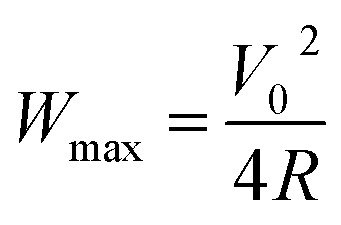
.

## 3 Results and discussion

### 3.1 Resistance components against electrode thickness *t*

**Fig. 2:**
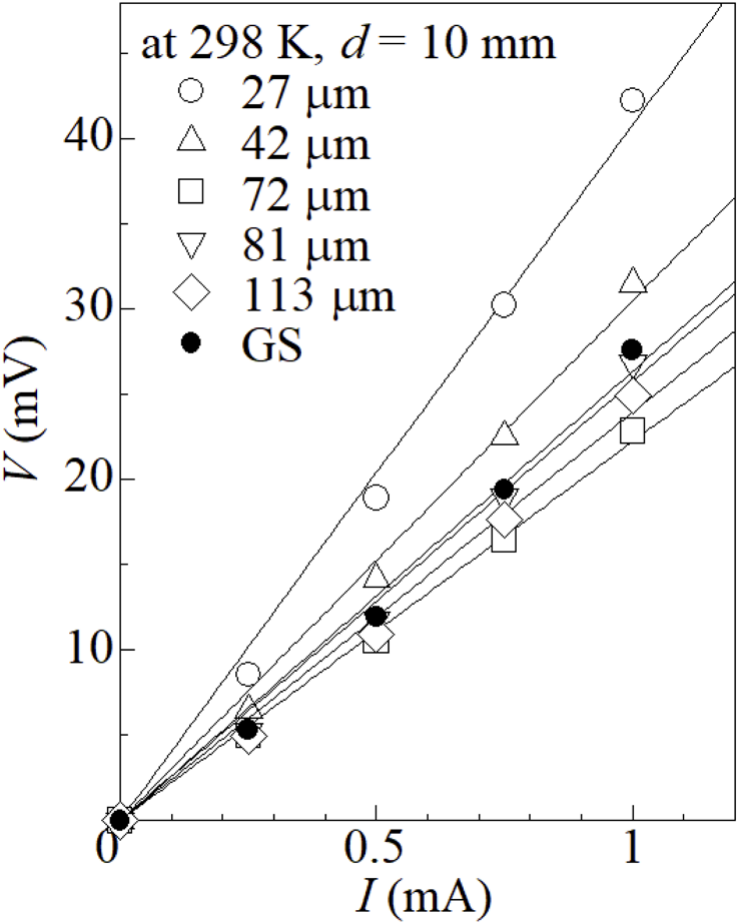
Voltage *V* against current *I* of LTEs composed of 0.8 M Na_4_[Fe(CN)_6_] and 0.8 M K_3_[Fe(CN)_6_] aqueous solution and graphite-dispersing electrodes at 298 K at different *t*. *s* and *d* were 42 mm^2^ and 10 mm, respectively. Filled circles are the data of LTE composed of commercially available GS (*t* = 220 μm). Straight lines are the results of least-squares fits.

Fig. 2 shows current *I*–voltage *V* plot of the ferro/ferri LTEs, which are composed of 0.8 M Na_4_[Fe(CN)_6_] and 0.8 M K_3_[Fe(CN)_6_] aqueous solution and graphite-dispersing electrode, at 298 K against *t*. *s* and *d* were 42 mm^2^ and 10 mm, respectively. At all temperatures, *V* increases in proportion to *I*. *R* was evaluated from the slope of the plot, as indicated by straight lines. With increase in *t*, *R* gradually decreases from 40.8 Ω at *t* = 27 μm to 22.4 Ω at 72 μm. With further increase in *t*, *R* slightly increases to 25.8 Ω at 81 μm and 24.0 Ω at 113 μm. Above *t* ≥ 40 μm, *R* becomes comparable with *R* of the LTE composed of GS.

**Fig. 3:**
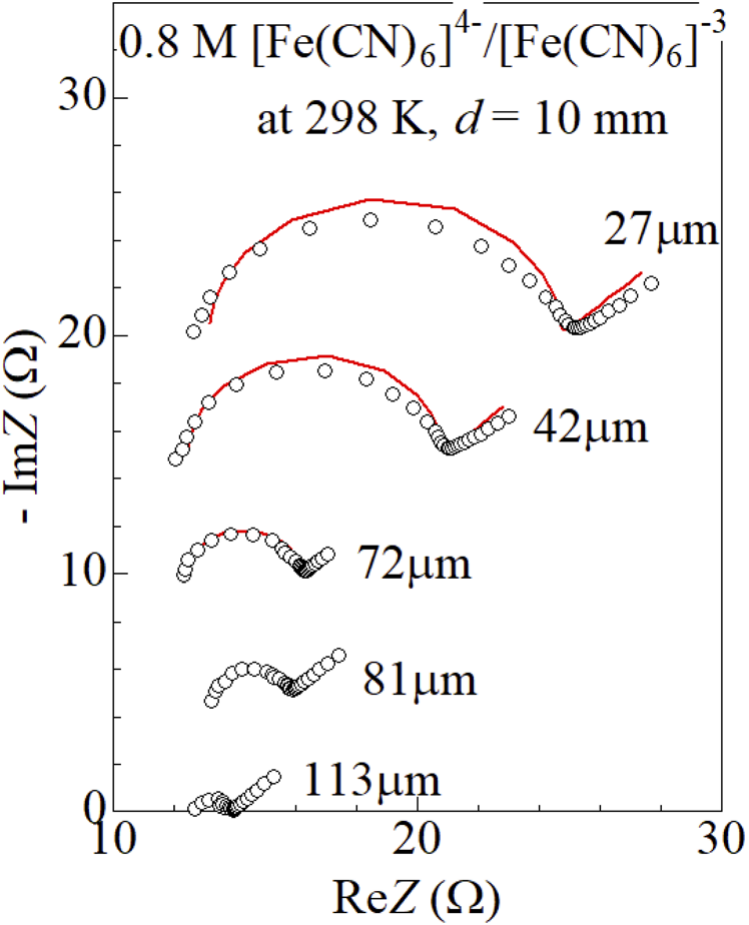
Nyquist plots of complex impedance of LTEs composed of 0.8 M Na_4_[Fe(CN)_6_] and 0.8 M K_3_[Fe(CN)_6_] aqueous solution and graphite-dispersing electrodes at 298 K at different *t*. The vertical axis is shifted for each plot. s and d were 42 mm^2^ and 10 mm, respectively. Solid curves are the results of least-squares fits with a Randles equivalent circuit composed of *R*_s_, *R*_ct_, *C*_d_, and *Z*_W_. The reliable factor 
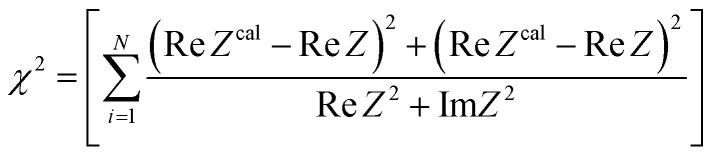
 was 8.5 × 10^−4^, 5.7 × 10^−4^, 1.6 × 10^−4^, 1.6 × 10^−4^, 1.1 × 10^−4^ at *t* = 27, 42, 72, 81, and 113 μm, respectively.

Fig. 3 shows Nyquist plots of complex impedance of the ferro/ferri LTEs at 298 K at different *t*. *s* and *d* were 42 mm^2^ and 10 mm, respectively. The vertical axis is shifted for each plot. The Nyquist plot at 27 μm shows a prototypical shape. The plot shows a semicircle at the left side and a straight line with an inclination of 45° at the right side. The resistances at the left and right sides of the semicircle correspond to *R*_s_ and *R*_s_ + *R*_ct_, respectively. Solid curves are the results of least-squares fits with a Randles equivalent circuit composed of *R*_s_, *R*_ct_, *C*_d_, and *Z*_W_ [Fig. 1(a)]. Feature of the observed impedance is well reproduced by the equivalent circuit. Similar behaviors are observed in the other Nyquist plots. *R*_s_, *R*_ct_, *C*_d_, and *A*_W_ were evaluated against *t* by least-squares fits with the Randles equivalent circuit We attempted to evaluate the parameters for GS, but was unable to obtain reliable values. We further evaluated 
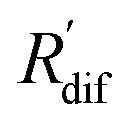
 by subtracting *R*_s_ + *R*_ct_ from *R*.

**Fig. 4:**
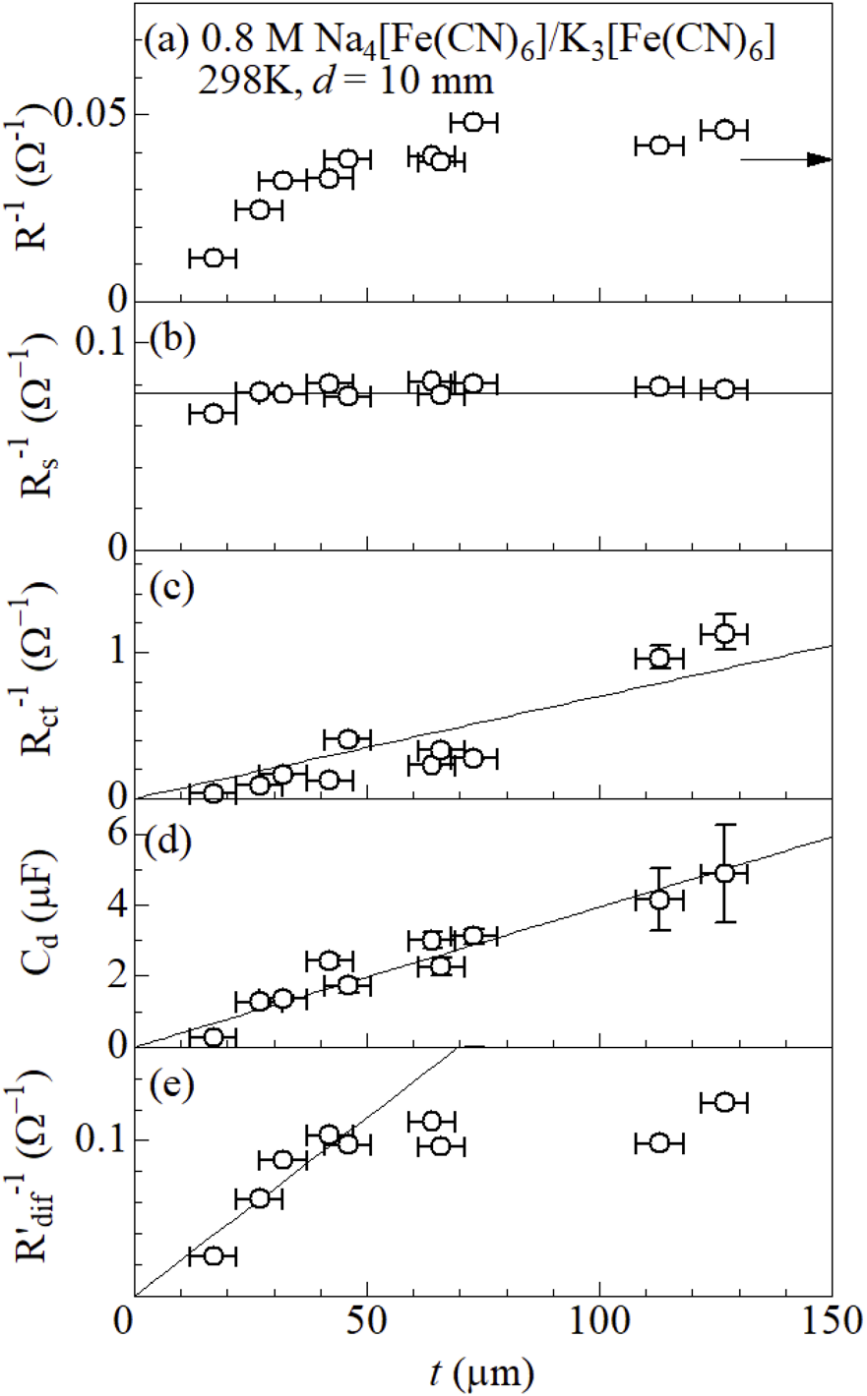
(a) *R*^−1^, (b) *R*_s_^−1^, (c) *R*_ct_^−1^, (d) *C*_d_, and (e) 
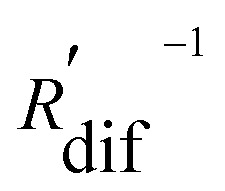
 of LTEs composed of 0.8 M Na_4_[Fe(CN)_6_] and 0.8 M K_3_[Fe(CN)_6_] aqueous solution and graphite-dispersing electrodes against *t* at 298 K. *s* and *d* were 42 mm^2^ and 10 mm, respectively. 
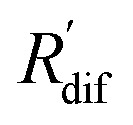
 was evaluated by subtracting *R*_s_ + *R*_ct_ from *R*. A horizontal arrow in (a) indicates *R*^−1^ of the LTE composed of GS (*t* = 220 μm). The solid straight lines in (b), (c), (d), and (e) are results of least-squares fit. The vertical error bars are those of *t*. The horizontal error bars in (b). (c), and (d) were those of *R*_s_, *R*_ct_, and *C*_d_, respectively. The errors were evaluated using equivalent circuit analyses. The horizontal error bars in (b) were smaller than the size of the symbols. The reliable factor 
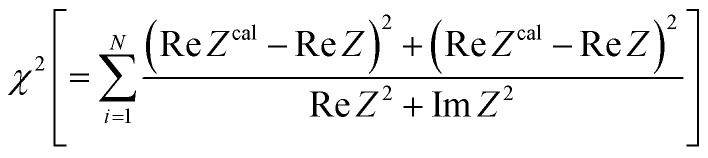
 was in the range of 1.1 × 10^−4^ to 8.5 × 10^−4^ except that (=2.8 × 10^−3^) at 17 μm.

Fig. 4(a) shows *R*^−1^ of the ferro/ferri LTEs against *t* at 298 K. In the small *t* region below 40 μm, *R*^−1^ steeply increases with *t*, and then nearly saturated at ≈ 0.04 Ω^−1^. The saturated value is almost the same as the *R*^−1^ value of GS. Fig. 4(b) shows *R*_s_^−1^ against *t* at 298 K. *R*_s_^−1^ (=0.076 Ω^−1^) is almost independent *d*, as indicated by straight line. This is because macroscopic electric force between the electrodes is independent of the microscopic electrode structure. A similar *t*-independent behavior of *R*_s_^−1^ is observed in the LTEs composed of 0.8 M Fe(ClO_4_)_2_ and 0.8 M Fe(ClO_4_)_3_ aqueous^27^ or methanol solutions.^28^Fig. 4(c) shows *R*_ct_^−1^ against *t* at 298 K. *R*_ct_^−1^ linearly increases with *t*, as indicated by straight line. The increase can be ascribed to the increase in EASA. Fig. 4(d) shows *C*_d_, which is a sensitive measure of EASA,^31–33^ against *t* at 298 K. As indicated by a straight line, *C*_d_ and hence EASA linearly increases with *t*.

Fig. 4(e) shows 
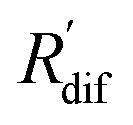
 against *t* at 298 K. Interestingly, 
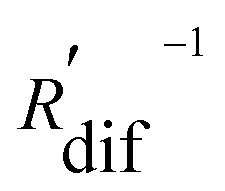
 linearly increases with *t* in the small *t* region (*t* ≤ 40 μm) while it becomes nearly constant in the thick *t* region. The increase in the thin *t* region is also ascribed to the increase in EASA [(e)]. Aiba *et. al.*^27^ reported a similar behavior of 
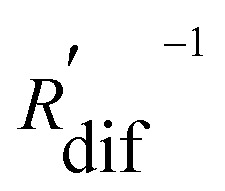
 in Fe^2+^/Fe^3+^ LTE. 
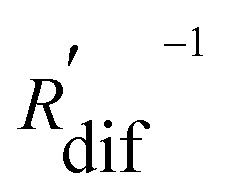
 linearly increases with *t* in the thin *t* region (*t* ≤ 80 μm) and becomes nearly constant in the thick *t* region. The saturation is probably ascribed to restricted mass transfer in a deep region of the electrode. In the deep region, mass transfer is too slow to continue the redox reaction. In other words, the graphite particles in the deep region are electrochemically inactive at the steady state and do not contribute to the Faraday current. In this scenario, characteristic thickness (*t*_ch_) where 
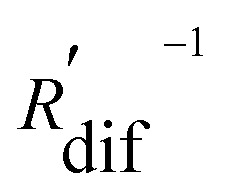
 begins to saturate corresponds to the minimum depth where graphite particles become inactive. It is reasonable that *t*_ch_ (=40 μm) of the ferro/ferri LTE is much smaller than *t*_ch_ (=80 μm) of the Fe^2+^/Fe^3+^ LTE, because the ionic radius of ferro/ferri (∼4.3 Å) is larger than that of Fe^2+^/Fe^3+^ (∼0.8 Å).

### 3.2 Low frequency EIS

In this subsection, we will quantitatively compare the resistance components of ferro/ferri LTE with the Fe^2+^/Fe^3+^ LTE, which is composed of aqueous solution containing Fe^2+^/Fe^3+^ redox couple. We note that the resistance components strongly depend not only on *d* and Δ*T*, but also on the electrolyte concentration and electrode material. To quantitatively compare the resistance components, the device parameters should be the same except for the type of solutes. We fabricated the Fe^2+^/Fe^3+^ LTE using the same cell and electrodes as used in the ferro/ferri LTE. The electrolyte was aqueous solutions containing 0.8 M Fe(ClO_4_)_2_ and 0.8 M Fe(ClO_4_)_3_. The solutes were purchased from FUJIFILM Wako corp. and used as received.

**Fig. 5:**
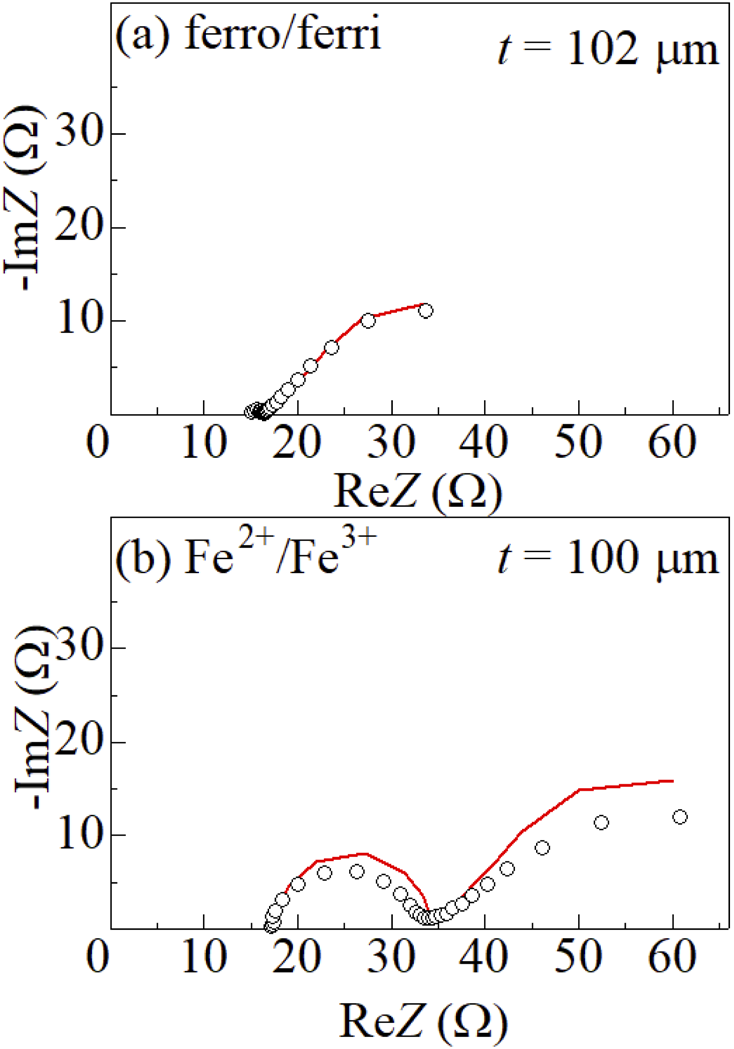
Nyquist plots of complex impedance of (a) ferro/ferri and (b) Fe^2+^/Fe^3+^ LTEs at 298 K. *s* and *d* were 42 mm^2^ and 10 mm, respectively. Solid curves are the results of least-squares fits with a Randles equivalent circuit composed of *R*_s_, *R*_ct_, *C*_d_, and 
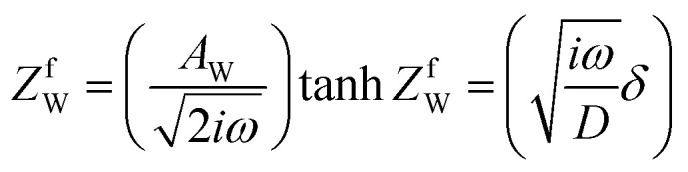
, where *δ*, and *D* are the thickness of the diffusion layer and diffusion constant, respectively. The reliable factor 
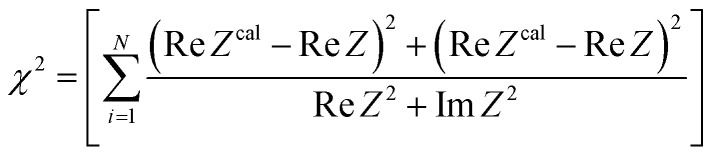
 was 2.9 × 10^−4^ and 2.0 × 10^−3^ for the ferro/ferri and Fe^2+^/Fe^3+^ LTEs, respectively.

Fig. 5 shows Nyquist plots of complex impedance of the (a) ferro/ferri and (b) Fe^2+^/Fe^3+^ LTEs at 298 K. The frequency range was expanded from 1 mHz to 25 kHz to directly evaluate *R*_dif_ by least-squares fit with a Randles equivalent circuit. *s* and *d* were 42 mm^2^ and 10 mm, respectively. The Nyquist plot of the (a) ferri/ferro LTE shows a prototypical shape. In the high *f* region (left region), the plot shows a semicircle at the left side and a straight line with an inclination of 45° at the right side. With further decreasing *f* (right region), the slope gradually becomes gentler and eventually becomes almost flat. The deviation from a 45-degree straight line in the low *f* region can be ascribed to the formation of the diffusion layer. The overall features are reproduced by a Randles equivalent circuit composed of *R*_s_, *R*_ct_, *C*_d_, and *Z*^*f*^_W_ [Fig. 1(b)], as indicated by solid curves. *R*_s_, *R*_ct_, *C*_d_, 
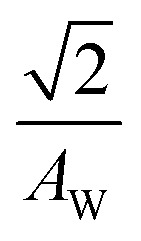
, and 
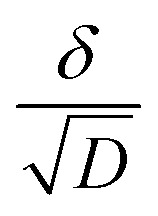
 were evaluated by least-squares fits with the Randles equivalent circuit and listed in Table 1. At low frequency limit (*ω* → 0), *Z*^f^_W_ converges a constant value 
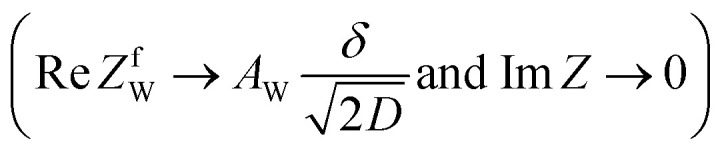
. *R*_dif_ corresponds to the low frequency limit of 
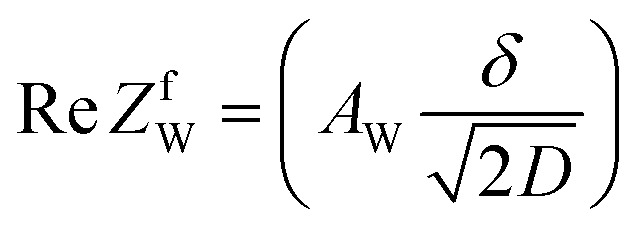
 and is also listed in Table 1.

**Table 1:** Parameters of ferro/ferri and Fe^2+^/Fe^3+^ LTEs at 0.8 M and at 298 K. *s* and *d* were 42 mm^2^ and 10 mm, respectively *R*_s_, *R*_ct_, *R*_dif_, *C*_d_, *A*_W_,and *R* are solution resistance, charge-transfer resistance, diffusion resistance, double-layer capacitance, Warburg coefficient, and device resistance, respectively. *R*_s_, *R*_ct_, *C*_d_, and 
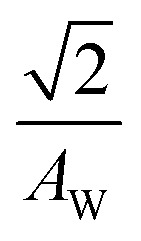
 were evaluated by least-squares fits with the Randles equivalent circuit composed of *R*_s_, *R*_ct_, *C*_d_, and 
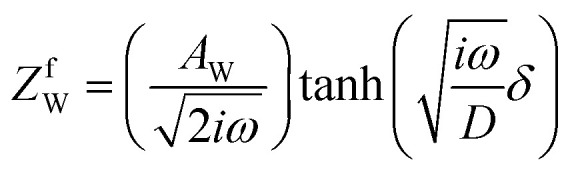
, where *δ*, and *D* are the thickness of the diffusion layer and diffusion constant, respectively) with fixing 
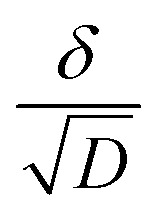
 at 14 s^1/2^ (ferro/ferri) or 13 s^1/2^ (Fe^2+^/Fe^3+^). *C*_d_ of ferro/ferri LTE was fixed at 4.9 μF due to the small semiciecle. The numbers in parentheses represent the errors evaluated by equivalent circuit analysis. 
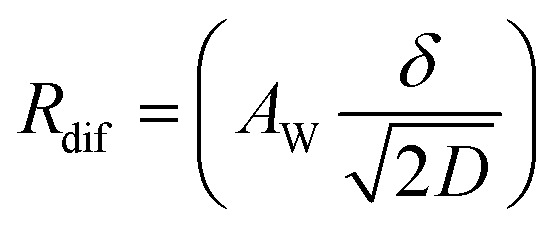
 is calculated by dividing 
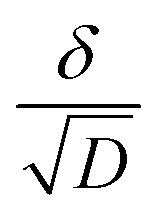
 by 
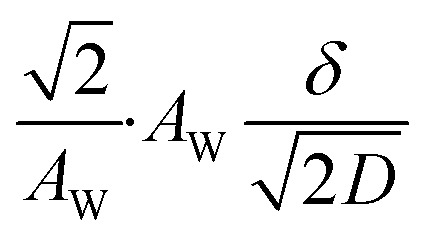
. *R* was evaluated by the slope of the *I*–*V* plot with use of DC current. 
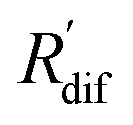
 was evaluated by subtracting *R*_s_ + *R*_ct_ from *R*

Redox couple	*t* (μm)	*R*_s_ (Ω)	*R*_ct_ (Ω)	*C*_d_ (μF)	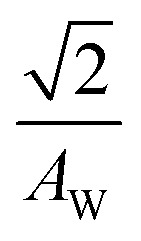 (s^1/2^/Ω)	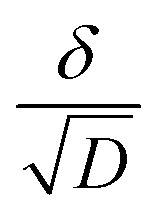 (s^1/2^)	*R*_dif_ (Ω)	*R* (Ω)	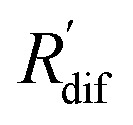 (Ω)
Ferro/ferri	102	15.2(5)	1.3(5)	4.9	0.494(7)	14	28.3	28.9	12.4
Fe^2+^/Fe^3+^	100	17.6(5)	16.4(8)	13.3(2.0)	0.333(16)	13	39.1	52.1	18.1

Similar behaviors are observed in (b) Fe^2+^/Fe^3+^ LTE. In Fe^2+^/Fe^3+^ LTE, the diameter of the semicircle is much larger than the corresponding diameter of the (a) ferro/ferri LTE. This indicates that *R*_ct_ of the Fe^2+^/Fe^3+^ LTE is much larger than that of the ferro/ferri LTE. In the Fe^2+^/Fe^3+^ LTE, the change in Re*Z* from the right side of the semicircle to the local maximum is ≈ 26 Ω while the corresponding change is ≈ 19 Ω in the ferri/ferro LTE. This suggests that *R*_dif_ of the Fe^2+^/Fe^3+^ LTE is larger than that of the ferro/ferri LTE. The solid curve in (b) is the least-squares fitted result with a Randles equivalent circuit composed of *R*_s_, *R*_ct_, *C*_d_, and *Z*^f^_W_. The obtained parameters were listed in Table 1. *R*_ct_ (=16.4 Ω) of the Fe^2+^/Fe^3+^ LTE is much larger than that (=1.3 Ω) of the ferro/ferri LTE. *R*_dif_ (=39.1 Ω) of the Fe^2+^/Fe^3+^ LTE is slightly larger than that (=28.3 Ω) of the ferro/ferri LTE. Therefore, the obtained parameters of the Fe^2+^/Fe^3+^ LTE are reliable even though the agreement between the observed data and the calculated values is not very good.

We will compare the resistance components between the ferro/ferri and Fe^2+^/Fe^3+^ LTEs. *R*_s_ (=15.2 Ω) of the former LTE is almost the same as that (=17.6 Ω) of the latter LTE. Interestingly, *R*_ct_ (=1.3 Ω) of the former LTE is much smaller than that (=16.4 Ω) of the latter LTE. This indicates that the redox reaction of [Fe(CN)_6_]^4−^/[Fe(CN)_6_]^3−^ is much faster than the redox reaction of Fe^2+^/Fe^3+^. *R*_dif_ (=28.3 Ω) of the former LTE is larger than that (=39.1 Ω) of the latter LTE. *R*_ct_ is an index of the difficulty of charge transfer at the electrode surface while *R*_dif_ is an index of the difficulty of mass transfer within the diffusion layer. The very small 
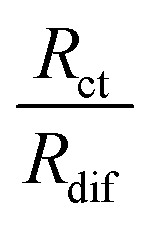
 (=0.08) in the ferro/ferri LTE suggests that mass transfer process is the main rate-limiting factor, probably reflecting large effective ionic radius of [Fe(CN)_6_]^4−^/[Fe(CN)_6_]^3−^ and resultant small *D*. In the steady state, there are few reactants at the electrode surface, and reactants transported by diffusion react quickly. On the other hand, the rather large 
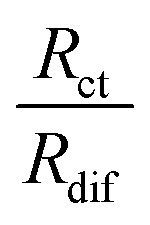
 (=0.42) in the Fe^2+^/Fe^3+^ LTE suggests both the charge and mass transfer processes play important roles in the steady state.

Finally, let us compare the diffusion resistance evaluated by two methods, *i.e.*, *R*_dif_ evaluated by EIS measurement and 
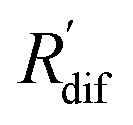
 evaluated by subtraction of *R*_s_ + *R*_ct_ from *R*. In Table 1, we listed *R* and 
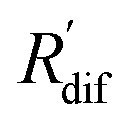
 (= *R* – *R*_s_ – *R*_ct_). For all LTEs, 
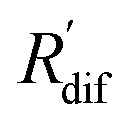
 is about half of *R*_dif_. This unexpected discrepancy between *R*_dif_ and 
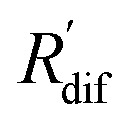
 is understandable if we consider the frequency response of *Z*^f^_W_. At sufficiently high *f* region (*f* ≫ 1), 
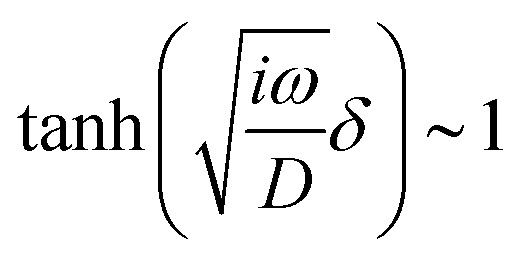
 and hence, *Z*^f^_W_ behaves the same as *Z*_W_. As *f* decreases to a characteristic frequency 
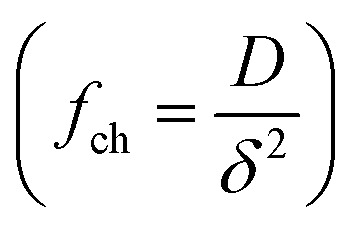
, *Z*^f^_W_ begins to deviate from *Z*_W_. With further decrease in *f*, the Nyquist plot of *Z*^f^_W_ show local maxima and then converges to a constant on the real axis; 
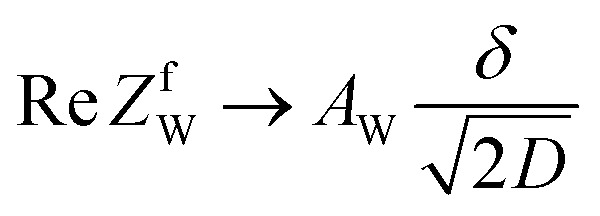
 and Im*Z* → 0 when *f* → 0. With use of the parameters shown Table 1, *f*_ch_ of the ferro/ferri and Fe^2+^/Fe^3+^ LTEs are estimated to be 5 and 6 mHz, respectively. To reach the ture stady state, LTE requires times several orders of magnitude longer than 
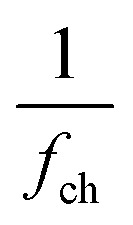
 (= several minutes). In the DC current measurement, the *V* value at each *I* was carefully measured after waiting several minutes until the time change of *V* almost disappeared. Several minutes, however, is too short for the LTE to reach the true steady state. It may take a few hours or tens of hours to reach the true steady state. In other words, *V* and hence 
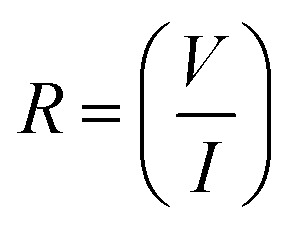
 is tends to be underestimated in the DC current measurement even if one measures *V* after waiting several minutes. Actually, *R* is roughly the same as the real part of each Nyquist plot at several mHz, whose inverse is several minutes. This is why 
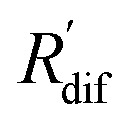
 is smaller than *R*_dif_. This argument indicates that absolute value of *R*_dif_ is more reliable than 
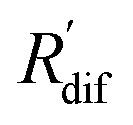
. Relative value of 
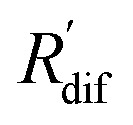
 may be reliable if 
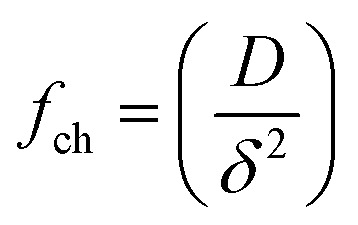
) is about the same.

**Fig. 6:**
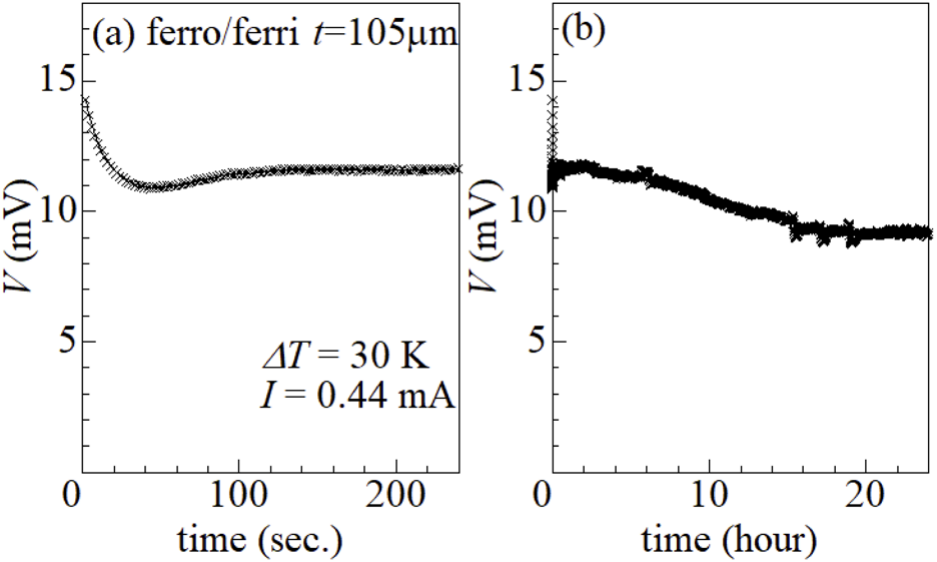
Time dependence of electromotive force *V* of ferro/ferri LTE (*t* = 105 μm) at Δ*T* = 30 K under constant current *I* of 0.44 mA; (a) short and (b) long time regions.

Fig. 6 shows time dependence of electromotive force *V* of ferro/ferri LTE (*t* = 105 μm) at Δ*T* = 30 K under constant current *I* of 0.44 mA. In (a) short time region, *V* rapidly decreases in the first several ten seconds and then becomes almost constant. With further increase in time [(b)], *V* decreases very slowly and finally becomes constant at ∼16 hours. The slow decrease in *V* can be ascribed slow formation of the diffusion layer, which reaches a steady-state at ∼16 hours. The rapid initial voltage drop can be ascribed to depletion of the reactants from the electrode surface due to the Faraday current. Looking closely at Fig. 6(a), one may notice that *V* shows a local minimum at 40 seconds and then slightly increases. This slight increase is probably due to onset of the diffusion process, which may be triggered by the depletion of the reactants.

### 3.3 Output characteristics

**Fig. 7:**
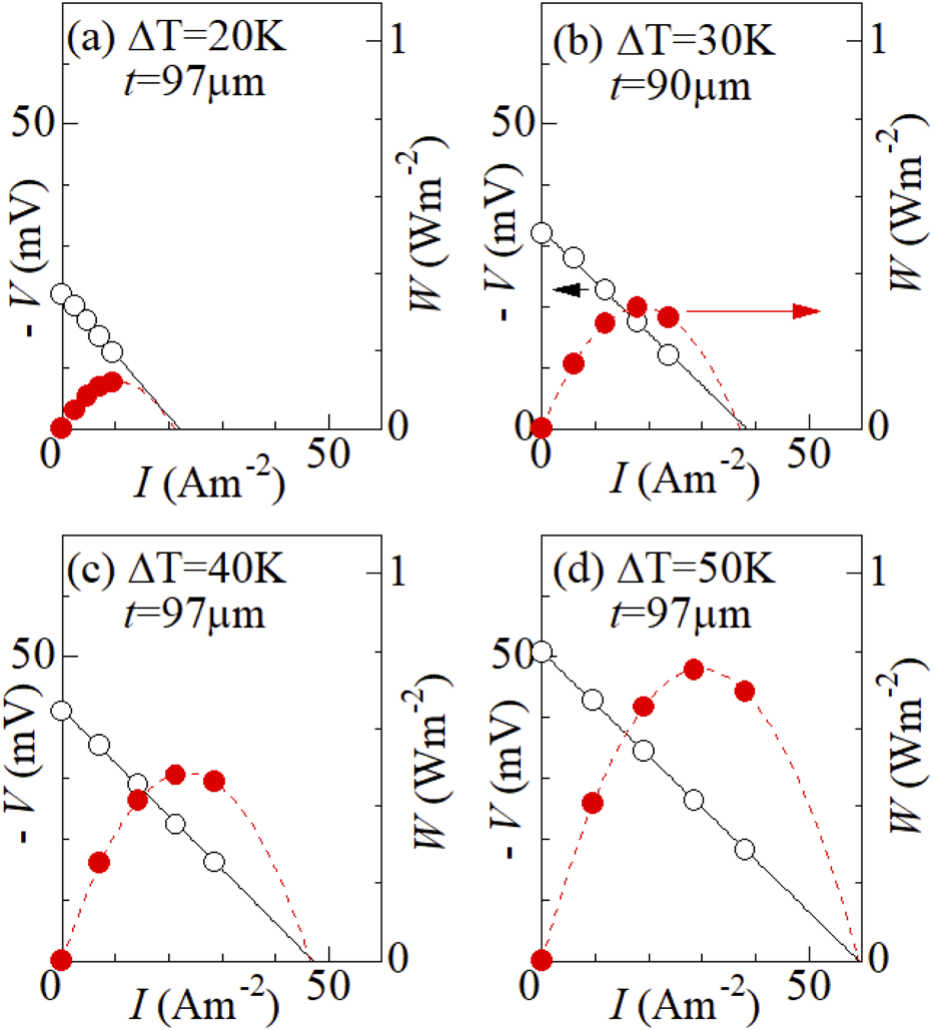
Output voltage *V* (open circles) and power density *W* (filled circles) against current density *I* of ferro/ferri LTEs at difference Δ*T*: (a) 20 K, (b) 30 K, (c) 40 K, and (d) 50 K. *s* and *d* were 42 mm^2^ and 10 mm, respectively. Solid straight lines are results of least-squares fits of the *I*–*V* plots. Broken curves are results of least-squares fits with quadratic function.

Fig. 7(a) shows output characteristics of the ferro/ferri LTEs at different Δ*T*. *d* and *s* were 10 mm and 42 mm^2^, respectively. Reflecting *R*, the *V* value linearly decreases with *I* as *V* = *V*_0_–*IR* (solid line). In Table 2, we listed thus evaluated *V*_0_, *R*, 
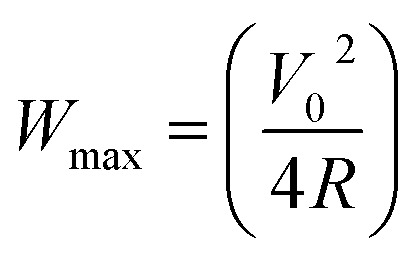
. *V*_0_ increases almost linearly with Δ*T*. *W*_max_ is expected to increase in proportion to the square of *V*_0_ (=*α*Δ*T*), and hence Δ*T*. As expected, *W*_max_ quadratically increases from 0.12 W m^−2^ at Δ*T* = 20 K, 0.31 W m^−2^ at Δ*T* = 30 K, 0.48 W m^−2^ at Δ*T* = 40 K, to 0.76 W m^−2^ at Δ*T* = 50 K. In Table 3, we compare thus obtained *W*_max_ with those of ferro/ferri LTEs reported in literature. We note that *R* strongly depend *d*, since *R*_s_ is proportional to *d* while *R*_ct_ and *R*_dif_ are independent on *d*.^27,28^ Furthermore, *W*_max_ increases in proportion to is proportional to Δ*T*^2^. Therefore, a direct comparison of *W*_max_ evaluated at different *d* and Δ*T* is difficult. Roughly speaking, however, *W*_max_ in the ferro/ferri LTE composed of the graphite-dispersing electrodes is comparable with those reported in literature.

**Table 2:** Output properties of ferro/ferri LTE. Δ*T*, *t*, *V*_0_, *R*, and 
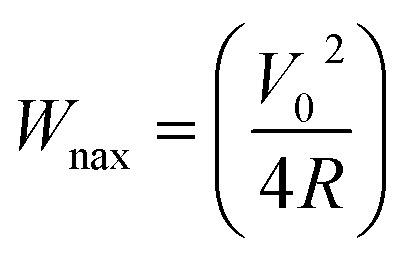
 are the temperature difference between the hot and cold electrodes, thickness of the electrode, open-circuit voltage, device resistance, and maximum output, respectively

Δ*T* (K)	*t* (μm)	*V*_0_ (mV)	*R* (mΩ m^−2^)	*W*_max_ (W m^−2^)
20	97	−22.3	1.01	0.12
30	90	−32.5	0.85	0.31
40	97	−41.3	0.88	0.48
50	97	−50.7	0.85	0.76

**Table 3:** Output characteristics of ferro/ferri LTEs. Δ*T*, *d*, *s*, *W*_max_, 
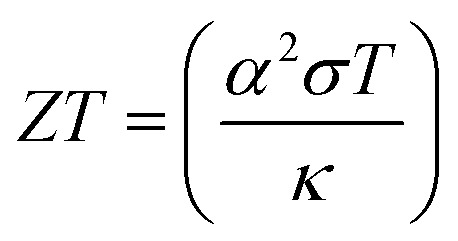
; *σ* and *κ* are electric and thermal conductivities, respectively), are the temperature difference, electrode distance, electrode area, maximum of output, and dimensionless figure of merit, respectively. In evaluation *ZT*, *T* was fixed at 298 K and *κ* was assumed to be 0.5 W Km^−1^

Δ*T* (K)	*d* (mm)	*s* (mm^2^)	*W*_max_ (W m^−2^)	*ZT*	References
22	20	512	0.05	0.005	8
20	5	100	0.47	0.014	9
10	15	—	0.10	0.036	10
10	10	42	0.01	0.002	17
20	10	42	0.12	0.007	This work
50	10	42	0.76	0.007	This work

The dimensionless figure of merit (
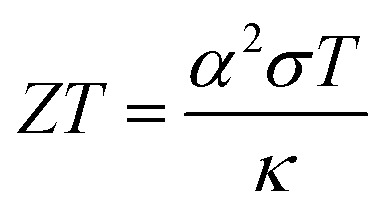
, *σ* and *κ* are electric and thermal conductivities, respectively) is a significant parameter for thermoelectric converter, since it determines the thermal efficiency *η*. Unlike *W*_max_, *ZT* does not depend on Δ*T*. *α* (=−1.04 mV K^−1^) was evaluated from the slop of the Δ*T* – *V*_0_ plot. 
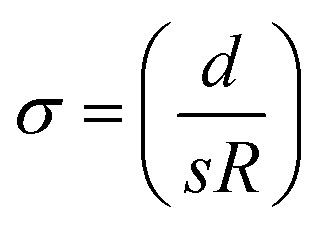
 was evaluated to be 0.11 S cm^−1^ using the average value (=0.90 Ω) of *R*. On the other hand, Kim *et al.*^9^ reported *κ* of aqueous solution containing *x* M K_4_[Fe(CN)_6_] and *x* M K_3_[Fe(CN)_6_] up to *x* = 0.4 M. We evaluated *κ* (≈0.5 W Km^−1^) at 0.8 M by extrapolation. Thus, we obtained *ZT* = 0.007 in the LTE composed of the graphite-dispersing electrode at 298 K. In Table 3, we compare thus obtained *ZT* with those of ferro/ferri LTEs reported in literature. We note that *R* strongly depends on *d*, since *R*_s_ is proportional to *d* while *R*_ct_ and *R*_dif_ are independent on *d*.^27,28^ The *ZT* values of our LTEs fall within the intermediate range among previously reported values.

## 4 Conclusions

We investigated the resistivity components in ferro/ferri LTE composed of graphite-dispersing electrodes against *t*. *R*_ct_^−1^ and *R*_dif_^−1^ linearly increase with *t* in the thin *t* region (*t* ≤ 40 μm) reflecting the increase in electrochemical active surface area (EASA). Analysis of the EIS data at low *f* reveals that mass transfer process is the main rate-limiting factor in ferro/ferri LTE. *W*_max_ of the ferro/ferri LTE (*t* = 97 μm) reaches 0.76 W m^−2^ at Δ*T* = 50 K, suggesting the effectiveness of the graphite-dispersing electrode in ferro/ferri LTE.

## Author contributions

Soshi Fukuda: data curation; formal analysis; investigation. Yutaka Moritomo: conceptualization; supervision; and writing – original draft; writing – review & editing.

## Conflicts of interest

There are no conflicts to declare.

## Data Availability

The data supporting this article will be provided if requested to the corresponding author.
